# Type 2 Diabetes Associated Changes in the Plasma Non-Esterified Fatty Acids, Oxylipins and Endocannabinoids

**DOI:** 10.1371/journal.pone.0048852

**Published:** 2012-11-08

**Authors:** Dmitry Grapov, Sean H. Adams, Theresa L. Pedersen, W. Timothy Garvey, John W. Newman

**Affiliations:** 1 Department of Nutrition, University of California Davis, Davis, California, United States of America; 2 Obesity and Metabolism Research Unit, United States Department of Agriculture - Agricultural Research Service - Western Human Nutrition Research Center, Davis, California, United States of America; 3 Department of Nutrition Sciences, University of Alabama at Birmingham and Birmingham VA Medical Center, Birmingham, Alabama, United States of America; Max Delbrueck Center for Molecular Medicine, Germany

## Abstract

Type 2 diabetes has profound effects on metabolism that can be detected in plasma. While increases in circulating non-esterified fatty acids (NEFA) are well-described in diabetes, effects on signaling lipids have received little attention. Oxylipins and endocannabinoids are classes of bioactive fatty acid metabolites with many structural members that influence insulin signaling, adipose function and inflammation through autocrine, paracrine and endocrine mechanisms. To link diabetes-associated changes in plasma NEFA and signaling lipids, we quantitatively targeted >150 plasma lipidome components in age- and body mass index-matched, overweight to obese, non-diabetic (n = 12) and type 2 diabetic (n = 43) African-American women. Diabetes related NEFA patterns indicated ∼60% increase in steroyl-CoA desaturase activity and ∼40% decrease in very long chain polyunsaturated fatty acid chain shortening, patterns previously associated with the development of nonalcoholic fatty liver disease. Further, epoxides and ketones of eighteen carbon polyunsaturated fatty acids were elevated >80% in diabetes and strongly correlated with changes in NEFA, consistent with their liberation during adipose lipolysis. Endocannabinoid behavior differed by class with diabetes increasing an array of N-acylethanolamides which were positively correlated with pro-inflammatory 5-lipooxygenase-derived metabolites, while monoacylglycerols were negatively correlated with body mass. These results clearly show that diabetes not only results in an increase in plasma NEFA, but shifts the plasma lipidomic profiles in ways that reflect the biochemical and physiological changes of this pathological state which are independent of obesity associated changes.

## Introduction

Obesity is a risk factor for the development of Type 2 diabetes, a disease which chronically increases circulating non-esterified fatty acids (NEFA) [1], dampens the pulsatile secretion of insulin [2], [3], and diminishes tissue glucose uptake while promoting hepatic glucose output [2], [4], [5]. Peripheral insulin resistance and fuel partitioning in type 2 diabetes are well-studied with respect to glucose, yet impacts on many metabolic domains remain to be assessed. Investigations of diabetes employing global metabolomics in plasma have reported changes in numerous metabolites including lipids, carbohydrates and amino acids, highlighting the fact that type 2 diabetes elicits broad perturbations of energy metabolism [6]–[8]. For example, diabetes increases circulating medium- and long-chain acylcarnitines [6] and branched-chain amino acids [7], [9], suggesting broad dysfunctions in fuel catabolism and mitochondrial function [6]. In contrast, studies addressing the impact of diabetes on circulating levels of low abundance signaling lipids including oxylipins (OxL) and endocannabinoids (eCBs) are less common. Here, we quantified many of these potent mediators along with NEFA to assess the covariant behavior of these molecules in obese diabetic and non-diabetic cohorts.

OxL and eCBs are structurally diverse groups of molecules with broad effects on cellular function acting through receptor- and ion channel-mediated processes [10], [11]. The arachidonic acid-derived OxLs (i.e. eicosanoids) are known to influence insulin signaling, inflammation and vascular function with mechanistic implications at the tissue level in diabetes and associated pathologies [10]. However, little is known regarding the impact of diabetes on plasma concentrations of eicosanoids and other polyunsaturated fatty acid (PUFA)-derived OxLs. Molecules with eCB properties include the N-acylethanolamides (NAEs), monoacylglycerols (MAGs) and lipoamino acids (LAA). Obesity and diabetes increase plasma and tissue arachidonate-derived eCBs, N-arachidonoylethanolamide (A-EA) and 2-arachidonylglycerol (2-AG) levels [11]. These changes are hypothetically linked to dysfunctions in eCB regulation involving dietary fat intake [12], leptin [13] and/or insulin signaling [14]. While distinct signaling and significant cross-talk exists between individual eCBs and their structural analogs [15] little is known regarding the impact of diabetes on the eCB array beyond A-EA and 2-AG. Moreover, while NEFA, OxL and eCB metabolism are linked [16], coordinated changes of these three pathways have not been previously characterized.

**Table 1:** Characteristics of overweight diabetic and non-diabetic African American Gullah-speaking female study participants.

Clinical Parameter	non-diabetic(n = 12)	type 2 diabetic(n = 43)
Body Mass (kg)	89.3±17	92.7±17
BMI, kg/m2 (range)	33±6 (24,43)	36±6 (26,47)
Age,yrs (range)	49±17 (21,69)	55±14 (19,87)
Glucose, mg/dL	92±10	210±79*
HbA1c (%)	5.4±1	9.1±2*
Lactate, mmol/L	1.1±0	1.2±0
Triglycerides, mg/dL	100±61	120±75
Cholesterol, mg/dL	200±52	210±47
HDL cholesterol, mg/dL	45±12	44±13
LDL cholestrol, mg/dL	140±42	140±41
VLDL cholesterol, mg/dL	20±12	23±15

* p<0.0001 by Mann-Whitney U-test.

To identify linkages between changes in NEFA and circulating signaling lipids, we performed a quantitative metabolomic investigation targeting over 150 plasma lipids including NEFA, OxLs and eCBs in overweight to obese, age and BMI matched non-diabetic and diabetic women. Multivariate analysis methods were used to identify key changes in biosynthetic relationships which are predictive of the diabetic phenotype.

## Methods

### Subjects and Study Design

Study volunteers were recruited in the Project SuGAR study, described in detail elsewhere [17]. This group displays an extraordinarily low genetic admixture, lives in a relatively small geographical space, and has a common dietary intake pattern which is uniformly rich in animal fats. There were 1279 registered participants in Project SuGAR, of which 290 were genotyped to identify persons with a missense uncoupling protein 3 (UCP3) G304A polymorphism. Of 52 subjects positive for the UCP3 g/a polymorphism (43 females, 9 males), complete datasets for clinical chemistries (blood lipids, glucose, lactate, HbA1c) and oral glucose tolerance test (OGTT) were available for 28 women (22 T2Ds, 6 non-diabetics). These subjects were thus chosen for subsequent metabolomics analyses, along with an age- and BMI-matched set of 28 women without the polymorphism (22 T2Ds, 6 non-diabetics) for comparison. A 2-way ANOVA was used to evaluate the association and interactions between the primary metabolic discriminates of type 2-diabetes reported below, and the UCP3 genotype. Neither significant associations with the UCP3 polymorphism nor diabetes x polymorphism interactions were detected (**Table S1**). A comparison of clinical parameters for subjects with and without type 2 diabetes is shown in **Table 1**. One diabetic subject was omitted due to insufficient sample volume. Of the 43 diabetic study participants included, 34 (79%) were on insulin and or a combination of oral anti-hyperglycemic and lipid lowering medications (insulin, n = 21; insulin and biguanide, n = 2; insulin, fibrate and thiazoladendiones, n = 1; biguanide, n = 2; sulfonylurea, n = 7; biguanide and sulfonylurea, n = 1).

**Table 2:** Plasma non-esterified fatty acids (µM) in BMI-matched obese non-diabetic and type 2 diabetic African-American women.*.

Lipid	non-diabetic(n = 12)	type 2 diabetic(n = 43)	ΔGM (%)
**Σ NEFA**	290 [109, 653]	621 [182, 1960]	114
**Σ SFA**	135 [47.0, 341]	281 [79.0, 787]	109
14∶0	2.93 [1.00, 19.0]	5.48 [1.00, 18.0]	87
16∶0	92.7 [30.0, 260]	196 [46.0, 582]	111
18∶0	38.7 [14.0, 66.0]	79.7 [30.0, 197]	106
19∶0	0.05 [0.02, 0.13]	0.09 [0.03, 0.37]	85
20∶0	0.12 [0.05, 0.43]	0.21 [0.01, 2.82]	75
**Σ MUFA**	54.8 [21.6, 124]	176 [48.0, 682]	220
**Σ n7 fatty acids**	8.76 [3.00, 28.0]	23.8 [8.00, 80.0]	172
**Σ n9 fatty acids**	46.0 [18.0, 95.0]	152 [40.0, 626]	230
16∶1n7	3.08 [1.00, 17.0]	8.78 [3.00, 38.0]	185
18∶1n7	5.68 [3.00, 10.0]	15.0 [5.00, 49.0]	165
18∶1n9	45.1 [18.0, 95.0]	150 [40.0, 620]	231
20∶1n9	0.37 [0.10, 0.65]	1.40 [0.14, 6.50]	278
**Σ PUFA**	99.2 [41.0, 185]	162 [33.0, 579]	63
18∶2n6	54.8 [24.0, 102]	103 [22.0, 363]	87
9ct,11t-CLA	0.85 [0.32, 2.00]	1.29 [0.34, 4.00]	52
18∶3n3	1.57 [0.34, 9.00]	4.02 [1.00, 16.0]	156
22∶4n6	0.42 [0.03, 2.00]	0.74 [0.05, 3.00]	76
22∶5n3	1.15 [0.12, 3.00]	2.17 [0.28, 9.00]	89
**Σ ** ***trans*** ** fatty acids**	44.7 [0.21, 3.65]	92.1 [0.86, 13.0]	106
trans 16∶1n7	0.76 [0.19, 2.50]	2.04 [0.50, 9.00]	168
trans 18∶2n6	0.28 [0.03, 1.00]	1.17 [0.05, 4.00]	211

* Values are reported as geometric means [ranges] if changes in geometric means between groups are significant (Mann-Whitney U-test, p<0.05 with FDR adjustment at q = 0.1). For remaining measurements see **Table S5**.

**Table 3:** Estimated enzyme activities in BMI-matched obese non-diabetic and type 2 diabetic African-American women based on plasma NEFA product to substrate ratios.*.

Activity Indices†	non-diabetic (n = 12)	type 2 diabetic (n = 43)	ΔGM (%)
**SCD**	0.60 [0.45, 1.01]	0.96 [0.29, 1.91]	60
16∶1n7/16∶0	0.03 [0.02, 0.07]	0.04 [0.02, 0.11]	35
18∶1n9/18∶0	1.16 [0.88, 1.98]	1.88 [0.56, 3.77]	62
**D6D**	0.08 [0.05, 0.13]	0.08 [0.05, 0.14]	–
18∶3n6/18∶2n6	0.01 [ND, 0.02]	0.01 [ND, 0.02]	–
20∶3n3/20∶4n6	0.15 [0.08, 0.25]	0.16 [0.10, 0.26]	–
**ELOVL2**	0.42 [0.24, 0.60]	0.72 [0.23, 10.4]	70
22∶4n6/20∶4n6	0.02 [ND, 0.04]	0.03 [ND, 0.19]	–
22∶5n3/20∶5n3	0.82 [0.47, 1.18]	1.40 [0.46, 20.8]	70
**ELOVL2/D6D/SPCS** ‡	6.71 [3.99, 25.2]	3.99 [1.48, 11.0]	−41
22∶5n6/22∶4n6	3.12 [0.96, 44.7]	1.37 [0.23, 17.0]	−56
22∶6n3/22∶5n3	8.76 [5.76, 26.2]	6.15 [2.71, 13.4]	−30

* Values are reported as geometric means [ranges] if changes in geometric means between groups are significant (Mann-Whitney U-test, p<0.05 with FDR adjustment at q = 0.1).

† SCD, steroyl-CoA desaturase; ELOVL2, elongase of very long chain fatty acids 2; D6D, delta 6 desaturase; SPCS, Sprecher pathway VLCPUFA chain shortening.

‡ ELOVL2/D6D/SPCS has inherent dependencies on other enzymes in the very long chain fatty acid synthesis pathway including ELOVL1 and ELOVL4.

**Figure 1:**
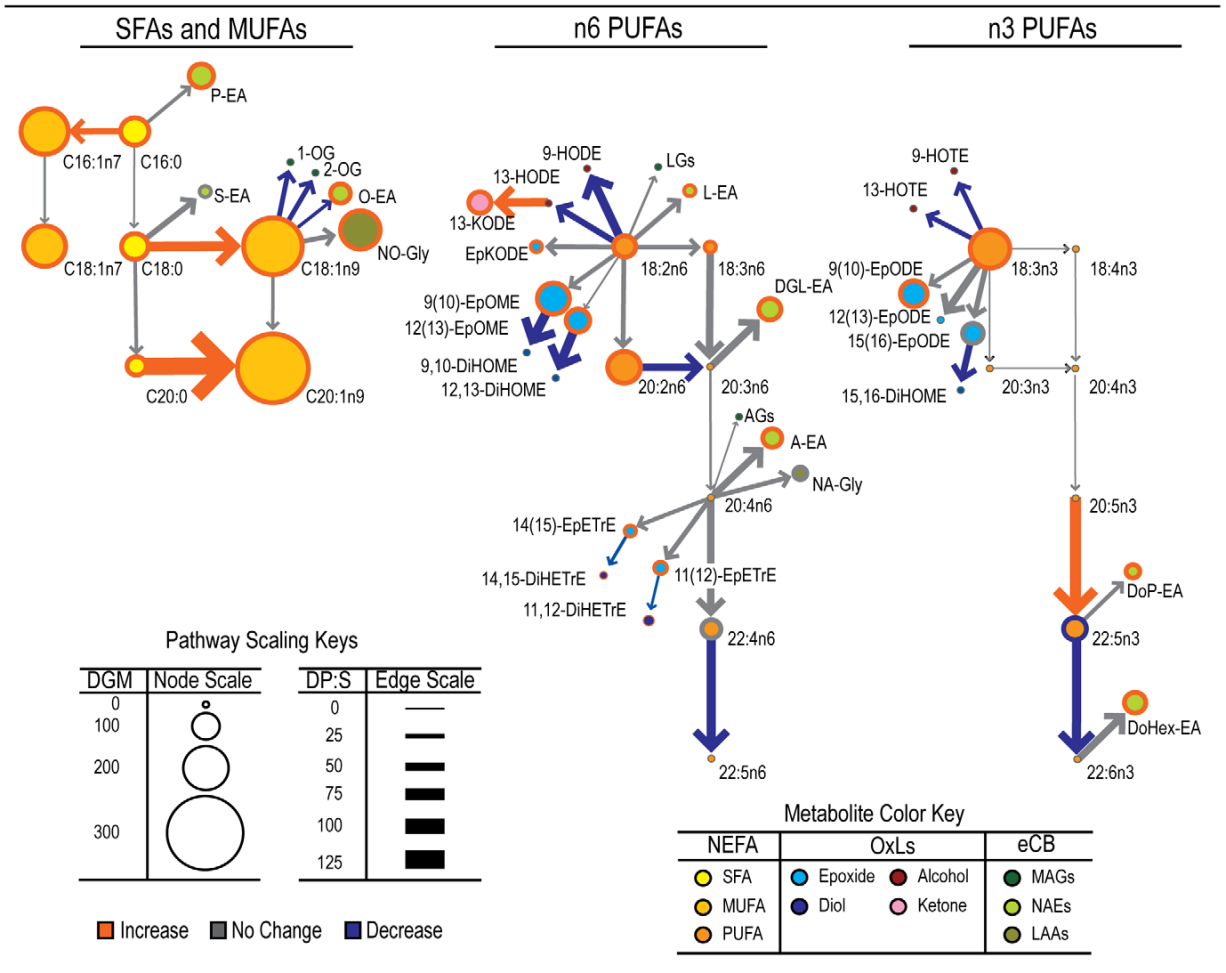
The type 2 diabetes-associated lipidomic changes projected in context of their biological relationships in obese African-American women. Metabolites are represented by circular “nodes” linked by “edges” with arrows designating the direction of the biosynthetic gradient (i.e. substrate to product). Some metabolites are linked by more than one enzymatic step. Node sizes represent magnitudes of differences in plasma metabolite geometric means (ΔGM). Arrow widths represent magnitudes of changes in product over substrate ratios (ΔP:S). Colors of node borders and arrows represent the significance and direction of changes relative to non-diabetics as per the figure legend. Differences are significant at p<0.05 by Mann-Whitney U test adjusted for FDR (q = 0.1).

The Institutional Review Boards of the University of California, Davis, Medical University of South Carolina, and the University of Alabama at Birmingham approved the studies and participants provided informed consent. Sample collection and plasma clinical chemistry analyses are described elsewhere [6]. Volunteers were instructed to eat their regular diets and avoid unusual activity or exercise for 3d prior to blood collection. Diabetic patients were instructed to refrain from oral medications the evening before and morning of the study. Patients treated with insulin (n = 23) were instructed to take regular or rapid-acting insulin at dinner the night before the study, but withhold intermediate- or long-acting insulin the evening before or morning of the blood collection. Blood was obtained between ∼0800 hrs and 0900 hrs by arm venipuncture into EDTA-treated tubes from fasted subjects: no food or drink since 2000 hrs the previous night. Plasma was frozen at −20°C for 1–7 d before transport to −80°C freezers. Plasma aliquots were used to carry out the current analysis, acylcarnitine profiling [6], and global metabolomics [7].

**Table 4:** Concentrations of selected plasma oxylipins (nM) in BMI-matched obese non-diabetic and type 2 diabetic African-American women.*.

Lipid	non-diabetic(n = 12)	type 2 diabetic(n = 43)	ΔGM (%)
**Σ oxylipins**	79.2 [43.4, 370]	91.1 [15.3, 365]	–
Σ**C18 Epoxides**	5.10 [2.12, 50.7]	9.4 [2.27, 60.6]	84
9(10)-EpODE	0.32 [0.10, 4.00]	0.66 [0.10, 6.00]	106
9(10)-EpOME	1.18 [0.30, 11.0]	2.68 [0.70, 20.0]	127
12(13)-EpOME	1.43 [0.50, 12.0]	2.76 [0.70, 17.0]	93
EKODE	1.69 [0.80, 18.0]	2.43 [0.20, 14.0]	44
Σ**C20 Epoxides**	1.14 [0.57, 5.14]	1.61 [0.48, 9.20]	41
11(12)-EpETrE	0.46 [0.20, 2.00]	0.70 [0.10, 4.00]	52
14(15)-EpETrE	0.24 [0.10, 1.00]	0.35 [0.10, 2.00]	46
Σ **Diols**	23.4 [9.50, 66.4]	22.4 [2.29, 71.9]	–
11,12-DiHETrE	0.44 [0.32, 0.70]	0.62 [0.34, 1.24]	41
14,15-DiHETrE	0.52 [0.38, 0.72]	0.67 [0.38, 1.44]	29
Σ **Ketones**	7.94 [4.05, 30.4]	13.2 [1.29, 75.7]	–
13-KODE	5.63 [3.00, 25.0]	10.5 [0.80, 68.0]	86

* Values are reported as geometric means [ranges] if changes in geometric means between groups are significant (Mann-Whitney U-test, p<0.05 with FDR adjustment at q = 0.1). For remaining measurements see **Tables S6** and **Table S7**.

### Oxylipins, Acylamides, Acylglycerols, and Lipoamino Acids Measurements

Oxylipins and eCBs were isolated and quantified using modifications of published protocols [18]. Briefly, plasma aliquots (100 µL) were spiked with deuterated OxL and eCB surrogates, and extracted with 60 mg Oasis HLB (Waters Corporation, Milford, MA) solid phase extraction cartridges. Solvents were removed under vacuum. Residues were reconstituted in methanol containing an internal standard, filtered at 0.1 µm and analyzed by UPLC-MS/MS. Analytes were separated with a 2.1×150 mm, 1.7 µm Acquity BEH column on an Acquity UPLC (Waters Inc, Milford MA), ionized by electrospray ionization and detected by multi-reaction monitoring on an API4000 QTRAP (AB-SCIEX, Foster City, CA). Oxylipins and NAEs/MAGs/LAAs were analyzed in independent injections and ionized in negative and positive modes, respectively. See **Table S2** for NAE, MAG and LAA mass transitions.

**Table 5:** Plasma N-acylamides and lipoamino acids (nM) in BMI-matched obese non-diabetic and type 2 diabetic African-American women.*

Lipid	non-diabetic (n = 12)	Diabetic (n = 43)	ΔGM (%)
Σ **NAE**	67.4 [27.0, 124]	106 [25.0, 440]	57
P-EA	9.59 [5.00, 40.0]	18.9 [6.00, 162]	97
O-EA	20.3 [8.00, 33.0]	36.2 [9.00, 175]	78
L-EA	7.91 [4.00, 27.0]	11.4 [5.00, 44.0]	44
DGL-EA	0.5 [0.30, 1.00]	0.93 [0.30, 4.00]	86
A-EA	2.09 [1.00, 3.00]	3.57 [1.00, 8.00]	71
DoP-EA	1.29 [0.20, 2.76]	1.97 [0.68, 4.66]	52
DoHex-EA	0.55 [0.30, 1.00]	1.02 [0.40, 2.00]	85
Σ **LAA**	8.67 [2.50, 36.1]	21.0 [4.50, 88.6]	142
NO-Gly	8.15 [2.00, 34.0]	20.2 [4.00, 86.0]	148

* Values are reported as geometric means [ranges] if changes in geometric means between groups are significant (Mann-Whitney U-test, p<0.05 with FDR adjustment at q = 0.1). For remaining measurements see **Table S8**.

### Non-esterified Fatty Acids (NEFA) Measurements

Plasma NEFA were isolated and converted to fatty acid methyl esters using a modified extractive methylation procedure [19]. Samples aliquots (25 µL) were spiked with 30 µL of 20 µM 15∶1n5 fatty acid (Nu-chek Prep, Inc., Elysian MN) in methanol, mixed with 125 µL ethereal diazomethane, and incubated for 10 min at 25°C. Solvents were removed by vacuum and residues were reconstituted in hexane containing internal standards and aliquots (1 µL) were analyzed on an HP6890 GC-5973N MSD (Agilent Technologies, San Jose, CA) equipped with a 30 m×0.25 id×0.25 µm DB-225 ms column (Agilent Technologies) with electron impact ionization. Spectral data was acquired in simultaneous selected ion monitoring/full scan (SIM/Scan) mode. Analytes were quantified with ChemStation vE.02.14 (Agilent Technologies) using internal standard methodologies against a 5 to 7 pt calibration curves.

### Data Quality Assurance and Control

Assay variability was assessed by analyzing sample replicates and laboratory reference materials in each batch and found to be stable across the study. Data was corrected for surrogate losses (see **Table S3**). Analytes are not reported if the signal to noise ratio is <2, calculated concentrations are below the lowest calibrant, or surrogate recoveries are below 40%. Of the ∼150 plasma lipids measured, 80 metabolites met these reporting criteria.

**Figure 2:**
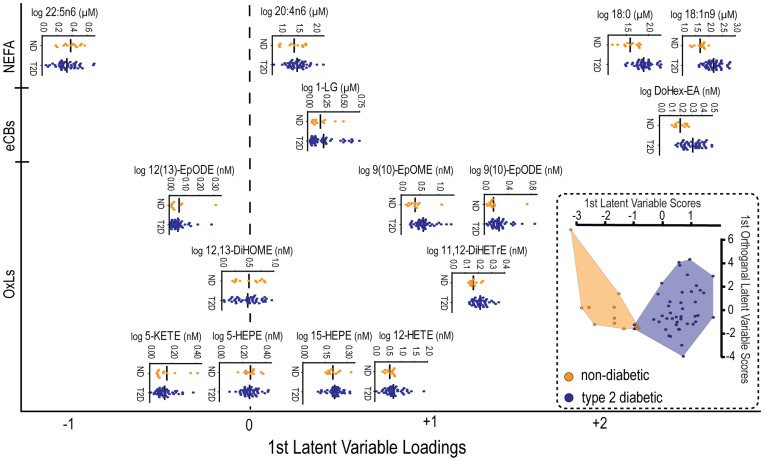
An OPLS-DA model built from 15 plasma lipids discriminates non-diabetic and diabetic cohorts. Horizontal scatter plots of the log transformed concentrations for each model variable are shown. The horizontal arrangement of metabolite scatter plots is scaled to their loading in the discriminant model. A given species importance in the classification increases with increasing displacement from the origin (broken line). The direction of the displacement, left or right, designates whether the species was decreased (left) or increased (right) in the diabetic relative to the non-diabetic patients. The overall model discrimination performance is presented as a scatter plot of subject model scores (inset).

**Figure 3:**
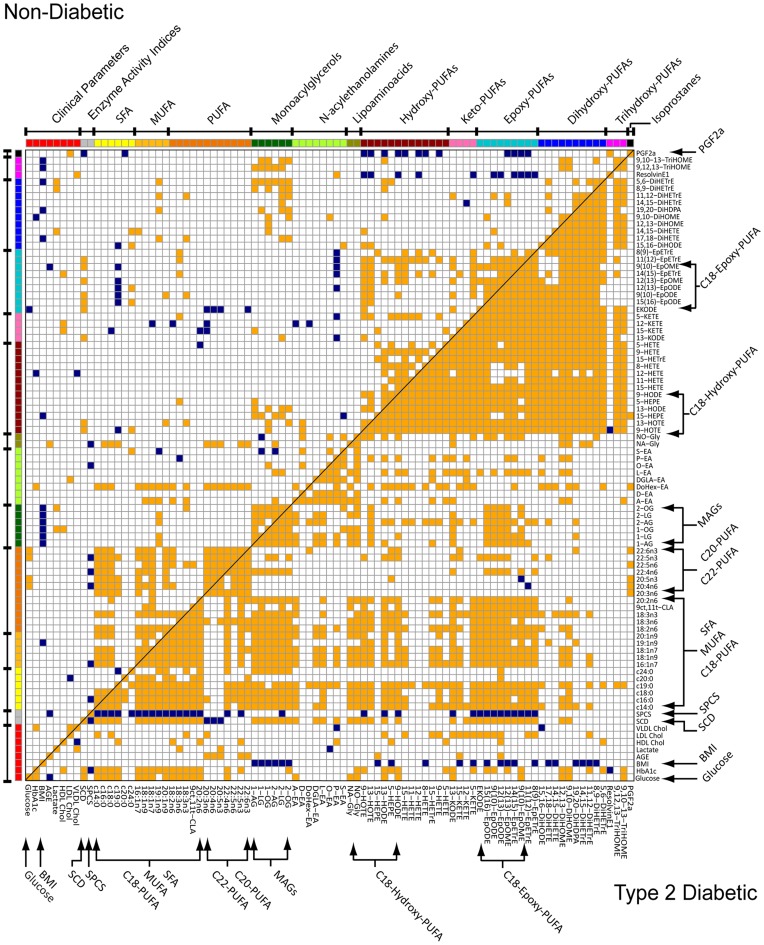
Analysis of correlations among all measured variables and estimated enzyme activities in non-diabetic and type 2 diabetic African-American women. Significant (p<0.05) non-parametric Spearman’s correlations for non-diabetic (top left triangle) and type 2 diabetic (bottom right triangle) subjects are indicated by orange (positive) and blue (negative) intersections.

**Figure 4:**
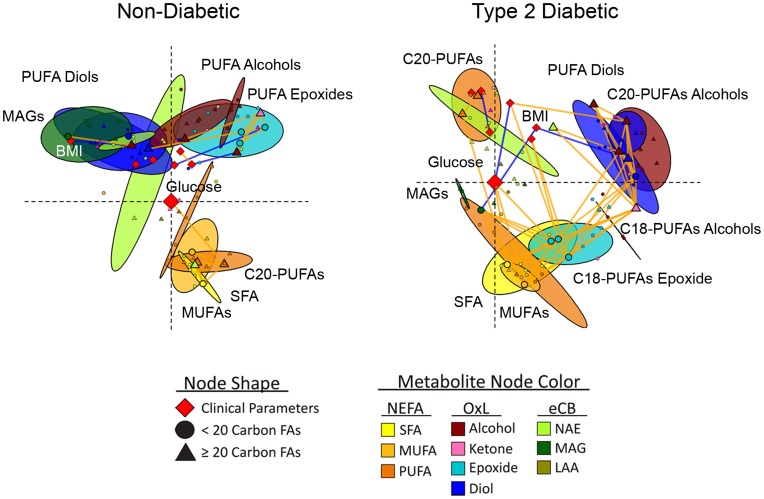
Parameter connectivity networks of metabolites and clinical parameters in African-American women with and without type 2 diabetes. Spearman’s correlations were used to generate multi-dimensionally scaled parameter connectivity networks for variable intercorrelations. Networks were oriented with fasting glucose at the origin and SFA in the lower right quadrant. Colored ellipses represent the 95% probability locations of metabolite classes (Hoettlings T^2^, p<0.05). Nodes indicate clinical parameters (diamonds), <20-carbon fatty acid metabolites (circles) and ≥20-carbon fatty acid metabolites (triangles), with discriminant model variables and glucose enlarged. Significant correlations between species are designated by orange (positive) or blue (negative) connecting lines (p<0.05, non-diabetic; p<0.01, diabetic participants).

### Univariate Statistics

Significant changes in metabolite concentrations and enzyme activity indices (i.e. product to substrate ratios) were assessed in R (version 2.13.1) [20] by Mann-Whitney U test. To control for the false discovery rate (FDR) associated with multiple hypothesis testing, the p<0.05 significance criterion was adjusted to allow a maximum 10% probability (q = 0.1) of false positive detection [21]. The percent change in geometric mean (GM) between diabetic and non-diabetic cohorts was calculated using the following equation:
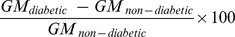



### Discriminant Modeling

Partial least squares projection to latent structures-discriminant analysis (PLS-DA) [22] and orthogonal PLS-DA (OPLS-DA) [23] multivariate classification models were developed for the study population. Model performance was optimized using a novel method for iterative variable selection (IVS). A metabolite-driven OPLS-DA model was compared to a model built with clinical variables alone [6]. The clinical parameters included plasma concentrations of glucose, lactate, HbA1c, triglycerides, and both total and lipoprotein cholesterol, as well as age, body mass, and BMI. Classification performance was determined by the goodness of the prediction statistic Q^2^ [23] and area under the receiver operator characteristic curve (AUROC).

The IVS feature selection approach belongs to a larger class of “wrapper methods” [24], and implements a heuristic (i.e. experience based) algorithm to accomplish a randomized, forward and backward variable subset selection. The full feature set is stochastically divided into subsets that undergo iterative backward variable deletion and forward variable addition until model performance converges to a local maxima, based on the cross-validated R^2^ or Q^2^. After each iteration the algorithm-optimized variable sets are combined and the procedure is repeated until the model performance converges to the global maxima. Due to the small study population feature selection was not included in the model validation procedures. However, the degree of model overfitting by this procedure was independently assessed.

IVS was implemented in R [package: pls [25]] using non-linear partial least squares [26] on log transformed and centered metabolite concentrations, scaled to unit variance. Using normality transformations optimized for each metabolite did not alter model results, as compared to uniformly log transformed data. The optimal model was chosen based on maximum Q^2^ from 100 independent IVS optimized PLS-DA models. To simplify model interpretation the selected model parameters were used to construct an OPLS-DA model, which collapses informative model aspects into a single latent variable. The degree of IVS optimized model overfitting was estimated by carrying out 7 independent IVS feature selection embedded model validations. The full data set was randomly split between training (2/3) and test sets (1/3). The training data was used to develop 100 IVS optimized sub-models. Comparison of these models’ predictive performances for the held out test sets to that of the training sets were used to estimate the degree of model overfitting for the reported model. Based on this investigation, the performance statistics of the final model shown in **Table S4** is expected to be lowered by ∼4±10% due overfitting.

OPLS-DA model training and validation was conducted in SIMCA-P+ v 12.0.1 (Umetrics; Umeå/Malmö, Sweden) by randomly assigning 2/3 of samples to training and remaining 1/3 to test sets. External OPLS-DA model cross-validation was conducted by repeating the training/testing procedure 3 times, to ensure that each subject was represented among one of the three test sets. The model diagnostic and performance statistics were calculated as the mean ± standard deviation of the 3 independent training/testing procedures. A summary model, including all subjects, was constructed to evaluate model scores and loadings. As a baseline for comparison, a clinical parameter OPLS-DA model (no IVS optimization) was generated using identical training and testing procedures.

### Parameter Connectivity Network Generation

Similarities in metabolite correlation patterns were translated into proximities within a visualized network using multi-dimensionally scaled Euclidean distances calculated from 1-|Spearman’s ρ|. Correlations between measurements (e.g. metabolites), represented by nodes (i.e. shapes), are designated by edges (i.e. connecting lines) whose characteristics are determined based on the Spearman’s rank-order coefficient of correlation (ρ) between two respective species. High density regions among biosynthetically or biologically related metabolites are highlighted by ellipses defined by Hotelling’s T^2^ (p<0.05) for group multivariate normal distributions within the coordinate space. Network visualizations were generated using imDEV v 1.4 [27].

## Results

### Plasma Free Fatty Acids

In subjects with Type 2 diabetes, increases in plasma NEFA and stearoyl-CoA desaturase (SCD) activity, and reductions in peroxisome-dependent synthesis of very long chain (≥22 carbon) PUFAs (VLCPUFAs) were observed. For instance, diabetic subjects showed a 114% increase in circulating NEFA (**Table 2**) and total NEFA was positively correlated with fasting glucose (ρ = 0.68, p<0.0001), with monounsaturated fatty acids (MUFA) showing the greatest magnitude of changes. The ratio of the MUFA species, palmitoleate (16∶1n7) and oleate (18∶1n9), to their saturated fatty acid (SFA) precursors, palmitate (16∶0) and stearate (18∶0), were used to estimate SCD activity [28]. As seen in **Table 3** and **Figure 1**, diabetes was associated with increases in both measures of SCD activity. A similar strategy was used to assess changes in VLCPUFA biosynthesis [29]. While the Δ6-desaturase (D6D) activity was unchanged in diabetic subjects, an increase in the elongase of very long chain fatty acids 2 (ELOVL2) activity is suggested by the ratio of 22∶5n3 over 20∶5n3 (**Table 3**). The conversions of 22∶4n6 to 22∶5n6 and 22∶5n3 to 22∶6n3 rely on the combined activities of ELOVL2, D6D and Sprecher pathway chain shortening (SPCS) via peroxisomal β-oxidation [30]. While not explicit defined within this calculation, this ratio may be further affected by other enzymes in the very long chain fatty acid biosynthesis which elongate 24 carbon fatty acids, including ELOVL1 and ELOVL4, to produce fatty acids up to 38 carbons in length. Since ELOVL2 increased and D6D was unchanged, changes in the ratios of 22∶5n6 over 22∶4n6 and 22∶6n3 over 22∶5n3 may suggest a reduction in SPCS activity, and/or a general reduction in very long chain biosynthesis in diabetic subjects. Changes in this potential marker of VLCPUFA biosynthesis activity were inversely correlated with the SCD activity index (ρ = −0.45; p = 0.002). Results for all measured plasma NEFA species can be found in **Table S5**.

### Plasma Free Oxylipins

While the total OxL concentration were unchanged in the diabetic state, increases in the concentrations of some fatty acid epoxides, diols and ketones were detected (Table 4). Each of the three measured linoleic acid (18∶2n6)-derived epoxides and one of three α-linolenic acid (18∶3n3)-derived epoxides were elevated from 47 to 127% in diabetic subjects (**Table 4**). In contrast two of the 3 measured arachidonic acid (20∶4n6)-derived epoxides, were elevated by ∼50%. The *vicinal* or 1,2-diol metabolites of the 18 carbon fatty acid epoxide were unchanged, while the arachidonate-derived 14,15- and 11,12-dihydroxyeicosatrieneoates (DiHETrE) were elevated by ∼35%. The fatty acid ketone, 13-KODE, an NAD+ dependent dehydrogenation product of the 18∶2n6 alcohol 13-HODE [31], was also elevated 86% in diabetic subjects. Analogous fatty acid ketones including 9-KODE and the arachidonate-derived KETEs either did not meet reporting criteria or did not display significant diabetes-associated effects (**Table S6** and **Table S7**).

### Plasma N-acylethanolamides and Lipoamino Acids

With the exception of stearoyl-ethanolamide, the measured NAEs were significantly elevated in diabetic subjects (**Table 5**). Of the two measured LAAs only N-oleoylglycine (NO-Gly) was elevated in diabetic subjects (**Table 5**). Mean MAG concentrations were unchanged in association with diabetes (**Table S8**) but showed negative correlations with BMI in these obese cohorts.

### Combined Analysis of NEFA, OxL and eCB Changes

The lipidomic changes associated with diabetes are projected in context of their biochemical relationships in **Figure 1**. Metabolites are represented by circular “nodes” with colored borders linked with “edges” represented by arrows designating the enzyme-dependent substrate to product transformation. Node color indicates chemical class while node size represents percent changes in metabolite concentrations. Edge widths represent percent changes in product to substrate ratios. The colors of node borders and edges indicate direction of significant changes, with gray indicating p>0.05.

In **Figure 1**, SFA and MUFA metabolites are displayed at the left, while omega-6 and omega-3 PUFA metabolites are displayed in the center and right. Orange circled nodes indicate increasing concentrations in diabetic subjects, with the low abundance eicosenoic acid (20∶1n9) showing the largest increase (276%; **Table 2**). As with 18∶1n9, N-oleoylethanolamide (O-EA) and NO-Gly also increased in diabetics. However, while the NO-Gly/18∶1n9 ratio was unchanged, the O-EA/18∶1n9 decreased as indicated by a blue connecting arrow. All NAEs (lime green nodes) matched the observed increases in their parent NEFA as shown by the linkage of these lipids by gray edges. This holds true for the epoxides (cyan nodes) as well. Unlike the diols derived from 20∶4n6 epoxides, diabetes-associated increases in the 18∶2n6- and 18∶3n3-derived epoxides are not matched by significant changes in their diols.

Changes in indices of enzymatic activity are also highlighted in this figure. Increased SCD activity is indicated by the orange arrows between all SCD-linked SFAs and MUFAs (e.g. 16∶0 to 16∶1n7 and 18∶0 to 18∶1n9 nodes), while the diabetes-associated decrease in SPCS activity is reflected in the blue arrows linking 22∶6n3 with 22∶5n3 and 22∶4n6 with 22∶5n6.

### Predictive Models for Type 2 Diabetes

OPLS-DA models built either with metabolites or clinical parameters were excellent predictors of type 2 diabetes. However, the metabolite-based model had superior classification statistics (Q^2^ = 0.61 vs. 0.46; AUROC = 0.97 vs. 0.94; **Table S4**) and highlighted metabolic shifts associated with diabetes. OPLS-DA model scores for each subject (**Figure 2**) correlate with both fasting glucose (r = 0.7, p<0.0001) and glycosylated hemoglobin (HbA1c; r = 0.5, p<0.05).

The model loading plot (**Figure 2**) describes the distributions and intercorrelations for model components in the context of their importance in the classification model. Metabolites are arranged based on their loading on the models’ predictive latent variable. Species with the greatest displacement from the origin (hashed line) indicate the most influential model parameters; whereas, the direction of their displacement designates if the metabolite was decreased (left) or increased (right) in diabetic compared to non-diabetic cohorts. The loading plot indicates that 18∶1n9, its precursor 18∶0, the docosahexaenoyl-ethanolamide (DoHex-EA) and the VLCPUFA 22∶5n6 are the dominant discriminating variables. Together, 18∶0 and 18∶1n9 report on the increased SCD activity in diabetic subjects. Changes in SFA but not MUFA are correlated with the VLCPUFA model components, 20∶4n6 and docosapentaenoate (22∶5n6), which are themselves highly correlated (p<0.0001) and report on the diabetes-associated change in VLCPUFA biosynthesis. The elevation in 18∶2n6- and 18∶3n3-derived epoxides 9(10)-EpOMEs and 9(10)-EpODEs displayed positive correlation with SFA and MUFA. The retention of the 20∶4n6 metabolite 12-HETE in the model is also of particular interest. While the 12-HETE values between diabetic and non-diabetic cohorts did not reach significance (p = 0.3), ∼20% of the diabetic subjects showed substantially elevated 12-HETE concentrations.

### Analysis of Intercorrelations Among Metabolites and Clinical Parameters

Correlation matrix heat maps (**Figure 3**) and parameter connectivity networks (PCNs; **Figure 4**) are used to visualize diabetes-associated changes in parameter relationships. Diabetes was associated with the striking emergence of metabolite and pathway correlations not apparent in the non-diabetic condition. Correlations among variables are designated by orange (positive) or blue (negative) heat map intersections (p<0.05) and PCN connecting lines (p<0.05, non-diabetic; p<0.01, diabetic). In **Figure 4** variables with similar intercorrelations are positioned closely, and metabolite classes are contained within ellipses representing their 95% probability regions. While powerful, this 3-dimensional projection can closely position uncorrelated compounds in the 2-dimensional display.

In short, variable intercorrelations are dramatically shifted in the diabetic state. The positive correlation between fasting glucose and C20-C22 PUFAs in the non-diabetic group is replaced by correlations with SFA, MUFAs, A-EA, and a selection of OxLs in the diabetic group. Conversely BMI is negatively correlated to MAGs in both groups, but negatively correlated with the 18 carbon epoxides and diols only in the diabetic cohort. In diabetes, LDL cholesterol gains a positive correlation with EpODEs and PUFA ketones (**Figure 3**). This display also highlights the shifts in the SCD and the VLCPUFA activity as reported by the SPCS activity indices. In the OPLS-DA model, DoHex-EA was the key acylethanolamide discriminant of diabetes. DoHex-EA is also positively correlated with SFA, MUFA, and PUFAs in the non-diabetic group only, a pattern not shared by A-EA (**Figure 3**). Finally, while OxL correlations are increased in diabetic subjects, class specific associations change. Notably, the 5-lipoxygenase-dependent products, 5-KETE and 5-HEPE increase their correlations with all PUFA-metabolites, while remaining uncorrelated to their parent lipids. In contrast, the isoprostanes became positively correlated with the >20 carbon PUFA pools in diabetic participants. These patterns of changes are well summarized in **Figure 4**, where the connectivity networks highlight the migration of epoxides (cyan ellipse) toward the bulk of the NEFAs (yellow and orange ellipses), while the >20 carbon PUFAs become dissociated from this group. Thus, the NEFA fine structure shifts from a highly intercorrelated matrix, having weaker correlations with the omega-3 PUFAs, to a clear subdivision between SFA/MUFA/18 carbon PUFAs and >20 carbon PUFAs.

## Discussion

The diabetes-associated perturbations of circulating lipid mediators and their relationships to plasma fatty acids are largely unexplored. Plasma NEFA concentrations reflect shift in NEFA uptake and release by adipose, liver and muscle [32], being dominated by adipose lipolysis in the fasted state [33]. Not unexpectedly, increases in plasma SFA and MUFA dominated the diabetes-associated changes in NEFA and were strong predictors of type 2 diabetes. However our targeted lipidomic survey uncovered subtle relationships between freely circulating NEFA, oxylipins, and endocannabinoids.

Based on the array of biosynthetically-related MUFA to SFA ratios, a net increase in systemic SCD activity was apparent in the diabetic cohort (**Table 3** and **Figure 1**). Increased SCD activity and/or expression have been reported in hypertriglyceridemia, obesity, nonalcoholic fatty liver disease (NAFLD), and the metabolic syndrome [34]–[37]. As the current study cohorts were BMI-matched and not hypertriglyceridemic [6], it is apparent that systemic SCD activity was elevated in the type 2 diabetic cohort beyond that expected by obesity alone. Such an observation is consistent with the increase in available glucoses, increasing de novo lipogenesis resulting in an elevation in SCD activity [35]

Despite the observed type 2 diabetes-associated increases in plasma NEFA, long and very long chain polyunsaturated fatty acids were not significantly elevated in this disease. In fact plasma markers of peroxisome-dependent VLCPUFA chain-shortening in both omega-3 and omega-6 pathways were reduced in diabetes, which also inversely correlated with SCD activity. Impaired VLCPUFA synthesis have previously been reported in the retina of streptozotocin-induced diabetic rats [38] and in NAFLD [39]. In fact, insulin resistance accelerates NAFLD in rodents [40] and observed changes in the VLCPUFA indices suggest an increased prevalence of NAFLD among the diabetic cohort.Diabetes and obesity are associated with pancreatic β-cell dysfunction, chronic inflammation, and vascular complications, conditions directly influenced by oxylipin metabolism [10]. While hyperglycemia and diabetes increase oxidative stress [41], and the total OxL concentration was positively correlated with fasting glucose, it did not increase in the diabetic compared to the non-diabetic cohort. Moreover, oxidative stress markers including the F2 isoprostanes were equivalent between cohorts. However, the concentration of multiple fatty acid epoxides, as well as several diols and ketones increased in association with diabetes. In particular, the epoxides of 18∶2n6 and 18∶3n3, and the 18∶2n6 ketone 13-KODE, were increased 2-fold and positively correlated with changes in SFA and MUFA. Changes in levels of 20∶4n6 and 20∶5n3-derived oxylipins were more subtle with ∼50% increases. Type 2 diabetes-associated changes in these species may arise from increased biosynthesis, decreased degradation, and or increased release of preformed metabolites. However, since fasting increases adipose lipolysis [33] and in this cohort epoxides and ketones are strongly correlated with plasma NEFA, we hypothesize that these species are derived from lipolysis of adipose. In support of this hypothesis, we have found that the adipose triglycerides of hamsters fed differing lipids in the diet preferentially accumulate 18 carbon species, with EpOMEs >5-fold over DiHOMEs, and KODEs ∼2-fold over HODEs in this pool [42].

Endocannabinoids and their related metabolites are important regulators of inflammation and energy balance, functioning through interaction with cannabinoid type 1 and type 2 receptors, transient receptor potential vanilloid type 1, and peroxisome proliferator activated receptors [11], [43], [44]. Previous investigations have reported increases in eCBs among both obese and diabetic humans [11]. Here plasma MAGs were inversely proportional to BMI, but unaffected by diabetes status. Conversely, plasma NAEs, including A-EA, were elevated and its concentrations correlated with SFA and MUFA in the diabetic cohort (**Figure 3**). Given that insulin dependent suppression of plasma A-EA is inversely correlated with liver fat [14], the higher A-EA in this studies diabetic cohort is consistent with insulin resistance and an increased prevalence of NAFLD. Similarly, increases in the omega-3 DoHex-EA were strong predictors for the type 2 diabetes-associate phenotype (**Figure 2**) and correlated with two 5-lipooxygenase metabolites, 5-HEPE and 5-KETE among diabetic participants. The basis for higher NAE metabolites in diabetes remains to be established, but an anti-inflammatory role is plausible. Specifically, the diabetes-associated elevation in SFA can activate Toll-Like Receptor 4 (TLR4), which has been reported to initiate inflammatory signaling and enhance NAE, but not MAG synthesis [45]. The NAEs may therefore act in a feedback loop to suppress inflammatory signaling through cannabinoid type 2 receptors [46].

In conclusion, to determine impacts of type 2 diabetes on the non-esterified plasma lipidome we quantified NEFA, oxylipins, acylethanolamides, lipoaminoacids and monoacylglycerides in weight-matched obese diabetic and non-diabetic cohorts. Diabetes-associated NEFA patterns indicate increases in SCD activity and decreases in VLCPUFA chain shortening, which may indicate impaired hepatic insulin sensitivity and/or fatty liver disease. Among diabetic participants, increases in 18 carbon epoxides and ketones correlate strongly with changes in SFA and MUFA, consistent with an enhanced release from adipose stores and/or suppressed degradation of these oxylipins. Type 2 diabetes is also associated with increases in NAEs and LAAs, but not MAGs. While the increases in NAE tone may constitute an adaptive mechanism to suppress inflammation developed in response to increases in circulating SFAs, MAG concentrations provide a diabetes-independent metabolic marker of body mass. Together, the observed changes in the plasma lipidome describe type 2 diabetes as a state of imbalance with respect to metabolic processes associated with fatty acid desaturation, VLCPUFA systhesis, adipose lipolysis, endocannabinoid tone and inflammatory activation. Oxylipin analysis revealed that these changes occur in the absence of systemic oxidative stress greater than that seen in obesity. Through this targeted lipidomic analysis, the following plasma markers were found as predictors of the type 2 diabetes phenotype: 1) 18∶1n9/18∶0, a marker of increased SCD activity; 2) 22∶5n6/22∶4n6, a marker of suppressed VLCPUFA synthesis; 3) 9(10)-EpOME and 9(10)-EpODE, putative markers of adipose lipolysis; 4) DoHex-EA, a marker of increased eCB system tone; 5) 1-LG, a metabolic marker of obesity.

## Supporting Information

Table S1 **Diabetes x UCP3 G304A polymorphism 2-way ANOVA p-values for Type 2 diabetes associated metabolic changes.** Type 2 Diabetes associated changes were not significantly different between evaluated UCP3 genotypes and no interactions were identified.
(DOC)

Table S2 **NAE, MAG and LAA mass transitions.** This table lists the positive mode electrospray ionization mass transitions used to detect and quantify the listed analytical targets.
(DOC)

Table S3 **Analytical surrogate recoveries.** The recovery and precision of analytical surrogates associated with non-esterified fatty acids (n = 1), endocannabinoids (n = 8) and oxylipins (n = 6) are reported.
(DOC)

Table S4 **Average modeling statistics for type 2 diabetes predictive models.** Orthogonal projection to latent structures discriminate analysis (OPLS-DA) models were constructed using either metabolites or clinical parameters. Both models accurately discriminated diabetic status, with the metabolite-based model slightly out-performing the clinical model in terms of model fit and the area under the receiver operator characteristic curve for model based prediction.
(DOC)

Table S5 **Plasma non-esterified fatty acids (µM) in obese African-American women.** Geometric mean and ranges are listed for all measured metabolites in this class.
(DOC)

Table S6 **Plasma eighteen carbon oxylipins (nM) in obese African-American women.** Geometric mean and ranges are listed for all measured metabolites in this class for experimental groups with and without Type 2 diabetes.
(DOC)

Table S7 **Plasma twenty and twenty-two carbon oxylipins (nM) in obese African-American women.** Geometric mean and ranges are listed for all measured metabolites in this class for experimental groups with and without Type 2 diabetes.
(DOC)

Table S8 **Plasma N-acylethanolamides (nM), lipoamino acids (nM) and monoacylglycerols (µM) in obese African-American women.** Geometric mean and ranges are listed for all measured metabolites in this class for experimental groups with and without Type 2 diabetes.
(DOC)
