# Supplementary material for: Type 2 Diabetes Associated Changes in the Plasma Non-Esterified Fatty Acids, Oxylipins and Endocannabinoids
Source: PLoS One. 2012 Nov 8;7(11):e48852. doi: 10.1371/journal.pone.0048852 (PMC3493609; doi:10.1371/journal.pone.0048852)
Supplement: Table S1 — Diabetes x UCP3 G304A polymorphism 2-way ANOVA p-values for Type 2 diabetes associated metabolic changes. Type 2 Diabetes associated changes were not significantly different between evaluated UCP3 genotypes and no interactions were identified. (DOC) [file pone.0048852.s001.doc]

Table S1: Diabetes x UCP3 G304A polymorphism 2-way ANOVA p‑values for Type 2 diabetes associated metabolic changes

| **Parameters** | **Type 2 Diabetes** | **UCP3**  **Genotype** | **Diabetes**  **x Genotype** |
| --- | --- | --- | --- |
| **Clinical Parameters** | | | |
| Glucose | <0.001 | 0.55 | 0.80 |
| **OPLS Parameters***^†^* | | | |
| LV | <0.001 | 0.68 | 0.53 |
| OLV | 1 | 0.89 | 0.80 |
| **OPLS-DA Model Variables** | | | |
| 18:1n9 | 0.002 | 0.43 | 0.65 |
| 18:0 | 0.001 | 0.69 | 0.68 |
| 20:4n6 | 0.46 | 0.31 | 0.65 |
| 22:5n6 | 0.37 | 0.77 | 0.74 |
| DoHex-EA | <0.001 | 0.37 | 0.88 |
| 1-LG | 0.38 | 0.42 | 0.85 |
| (9)10-EpODE | 0.35 | 0.56 | 0.18 |
| (9)10-EpOME | 0.14 | 0.66 | 0.32 |
| (12)13-EpODE | 0.55 | 0.72 | 0.33 |
| (11)12-DiHETrE | 0.057 | 0.28 | 0.97 |
| (12)13-DiHOME | 0.74 | 0.18 | 0.67 |
| 12-HETE | 0.25 | 0.28 | 0.53 |
| 15-HEPE | 0.53 | 0.22 | 0.83 |
| 5-HEPE | 0.93 | 0.53 | 0.24 |
| 5-KETE | 0.39 | 0.95 | 0.55 |
| **Activity Indices*^‡^*** | | | |
| SCD | 0.001 | 0.57 | 0.66 |
| SPCS | 0.002 | 0.90 | 0.77 |
| D6D | 0.79 | 0.063 | 0.89 |
| ELOVL2 | 0.20 | 0.63 | 0.86 |

† – LV, predictive latent variable subject scores, OLV, first orthogonal latent variable subject scores.

‡ – SCD, steroyl-CoA desaturase; ELOVL2, elongase of very long chain fatty acids 2; D6D, delta 6 desaturase; SPCS, Sprecher pathway VLCPUFA chain shortening.
