# Supplementary material for: Type 2 Diabetes Associated Changes in the Plasma Non-Esterified Fatty Acids, Oxylipins and Endocannabinoids
Source: PLoS One. 2012 Nov 8;7(11):e48852. doi: 10.1371/journal.pone.0048852 (PMC3493609; doi:10.1371/journal.pone.0048852)
Supplement: Table S2 — NAE, MAG and LAA mass transitions. This table lists the positive mode electrospray ionization mass transitions used to detect and quantify the listed analytical targets. (DOC) [file pone.0048852.s002.doc]

Table S2: NAE, MAG and LAA mass transitions.

| **Compound** | **Abbreviation** | **Precursor Ion**  **(m/z)** | **Product Ion**  **(m/z)** |
| --- | --- | --- | --- |
| **d8-Arachidonoyl-EA***^†^* | **d8-A-EA** | **356.3** | **63.1** |
| Palmitoyl-EA | P-EA | 300.2 | 62.1 |
| α-linolenoyl-EA | AL-EA | 322.2 | 62.1 |
| Linoleoyl-EA | L-EA | 324.2 | 62.1 |
| Stearoyl-EA | S-EA | 328.2 | 62.1 |
| Oleoyl-EA | O-EA | 326.2 | 62.1 |
| Dihomo-γ-linoleoyl-EA | DGL-EA | 350.3 | 62.1 |
| Arachidonoyl-EA | A-EA | 348.3 | 62.1 |
| Docosatetraenoyl-EA | DoTet-EA | 376.3 | 62.1 |
| Docosahexenoyl-EA | DoHex-EA | 372.3 | 62.1 |
| **d5-2-Arachidonoyl-glycerol** | **d5-2-AG** | **384.3** | **287.2** |
| 1-Oleoyl-glycerol | 1-OG | 357.3 | 265.2 |
| 2-Oleoyl-glycerol | 2-OG | 357.3 | 265.2 |
| 1-Linoleoyl-glycerol | 1-LG | 355.3 | 263.2 |
| 2-Linoleoyl-glycerol | 2-LG | 355.3 | 263.2 |
| 1-Arachidonoyl-glycerol | 1-AG | 379.3 | 287.2 |
| 2-Arachidonoyl-glycerol | 2-AG | 379.3 | 287.2 |
| **d8-N-Arachidonyl-glycine** | **d8-NA-Gly** | **370.3** | **76.1** |
| N-Oleoyl-glycine | NO-Gly | 340.2 | 76.2 |
| N-Arachidonyl-glycine | NA-Gly | 362.3 | 76.1 |

*†* - EA, ethanolamide.
