# Supplementary material for: Type 2 Diabetes Associated Changes in the Plasma Non-Esterified Fatty Acids, Oxylipins and Endocannabinoids
Source: PLoS One. 2012 Nov 8;7(11):e48852. doi: 10.1371/journal.pone.0048852 (PMC3493609; doi:10.1371/journal.pone.0048852)
Supplement: Table S3 — Analytical surrogate recoveries. The recovery and precision of analytical surrogates associated with non-esterified fatty acids (n = 1), endocannabinoids (n = 8) and oxylipins (n = 6) are reported. (DOC) [file pone.0048852.s003.doc]

Table S3: Analytical surrogate recoveries

| **Chemical Class** | **Compound** | **Mean ± sd** | **%RSD*^†^*** |
| --- | --- | --- | --- |
| Fatty Acid (FA) | 15:1n5 | 53 ± 13 % | 25% |
| prostamide | d4-PGF2a-EA | 76 ± 14 % | 19% |
| N-acylethanolamide | d8-A-EA | 58 ± 31 % | 53% |
| monoacylglygerol | d5-2-AG | 83 ± 32 % | 38% |
| lipoaminoacid | d8-NA-Gly | 87 ± 42 % | 48% |
| prostanoid prostanoidprostanoidostaglandins | d4 6-keto PGF1a | 46 ± 17 % | 36% |
| thromboid | d4-TXB2 | 60 ± 12 % | 20% |
| prostanoid | d4-PGE2 | 50 ± 13 % | 26% |
| prostanoid | d4-PGD2 | 55 ± 10 % | 18% |
| FA diol | d11-14,15-DiHETrE | 81 ± 15 % | 18% |
| FA primary alcohol | d6-20-HETE | 68 ± 12 % | 18% |
| FA secondary alcohol | d4-9(S)-HODE | 65 ± 15 % | 22% |
| FA secondary alcohol | d8-12(S)-HETE | 62 ± 10 % | 16% |
| FA secondary alcohol | d8-5(S)-HETE | 69 ± 17 % | 25% |
| FA epoxide | d8-11(12)-EpETrE | 76 ± 18 % | 24% |

*†* -Relative standard deviation (standard deviation divided by the mean) x 100
