# Supplementary material for: Type 2 Diabetes Associated Changes in the Plasma Non-Esterified Fatty Acids, Oxylipins and Endocannabinoids
Source: PLoS One. 2012 Nov 8;7(11):e48852. doi: 10.1371/journal.pone.0048852 (PMC3493609; doi:10.1371/journal.pone.0048852)
Supplement: Table S4 — Average modeling statistics for type 2 diabetes predictive models. Orthogonal projection to latent structures discriminate analysis (OPLS-DA) models were constructed using either metabolites or clinical parameters. Both models accurately discriminated diabetic status, with the metabolite-based model slightly out-performing the clinical model in terms of model fit and the area under the receiver operator characteristic curve for model based prediction. (DOC) [file pone.0048852.s004.doc]

Table S4: Average modeling statistics for type 2 diabetes predictive models*

| **OPLS-DA Models** | **Variables** | **LV ^†^** | **R^2^X ^‡^** | **R^2^Y ^§^** | **Q^2^ ||** | **AUROC ^¶^** |
| --- | --- | --- | --- | --- | --- | --- |
| Metabolite-based ^#^ | 15 | 1(2) | 0.52 ± 0.1 | 0.71 ± 0.01 | 0.61 ± 0.1 | 0.97 |
| Clinical-based ** | 11 | 1(2) | 0.53 ± 0.1 | 0.66 ± 0.1 | 0.46 ± 0.1 | 0.94 |

***** - Reported model statistics are the mean ± SD of three training and testing iterations

† *-* Number of predictive and (total) latent variables

‡ *-* Variance in parameters explained by model

§ *-* Variance in class assignment explained by model

|| *-* Discriminatory capability (fit) of the model

¶ *-* Area under the receiver operator characteristic curve for model based predictions

# *-* Model comprised of IVS selected metabolites

** *-* Model comprised of plasma concentrations of glucose, lactate, HbA1c, triglycerides, and both total and lipoprotein cholesterol, as well as age, body mass, and BMI (6).
