# Supplementary material for: Type 2 Diabetes Associated Changes in the Plasma Non-Esterified Fatty Acids, Oxylipins and Endocannabinoids
Source: PLoS One. 2012 Nov 8;7(11):e48852. doi: 10.1371/journal.pone.0048852 (PMC3493609; doi:10.1371/journal.pone.0048852)
Supplement: Table S5 — Plasma non-esterified fatty acids (µM) in obese African-American women. Geometric mean and ranges are listed for all measured metabolites in this class. (DOC) [file pone.0048852.s005.doc]

**Table S5: Plasma non-esterified fatty acids (μM) in obese African-American women *^†^***

| **Lipid** | **non-diabetic**  **(n=12)** | | **T2D**  **(n=43)** | |
| --- | --- | --- | --- | --- |
| **SFA** |  |  |  |  |
| 14:0 | 2.93 | [1.00, 19.0] | 5.48 | [0.83, 18.0] |
| 16:0 | 92.7 | [30.0, 260] | 196 | [46.0, 582] |
| 18:0 | 38.7 | [14.0, 66.0] | 79.7 | [30.0, 197] |
| 19:0 | 0.05 | [0.02, 0.13] | 0.09 | [0.02, 0.37] |
| 20:0 | 0.12 | [0.05, 0.43] | 0.21 | [0.01, 3.00] |
| 24:0 | 0.15 | [0.04, 0.35] | 0.13 | [0.01, 0.94] |
| **MUFA** |  |  |  |  |
| 16:1n7 | 3.08 | [0.68, 17.0] | 8.78 | [3.00, 38.0] |
| 18:1n9 | 45.2 | [18.0, 95.0] | 150 | [39.0, 620] |
| 18:1n7 | 5.68 | [3.00, 10.0] | 15.0 | [5.00, 49.0] |
| 19:1n9 | 0.50 | [0.08, 2.00] | 0.84 | [0.17, 4.00] |
| 20:1n9 | 0.37 | [0.10, 0.65] | 1.40 | [0.14, 6.00] |
| **PUFA** |  |  |  |  |
| 18:2n6 | 54.8 | [24.0, 102] | 103 | [22.0, 363] |
| 18:3n6 | 0.47 | [0.09, 2.00] | 0.68 | [0.04, 3.00] |
| 18:3n3 | 1.57 | [0.34, 9.00] | 4.02 | [0.60, 16.0] |
| 9ct,11t-CLA | 0.85 | [0.32, 2.00] | 1.29 | [0.34, 4.00] |
| 20:2n6 | 0.66 | [0.04, 2.00] | 1.52 | [0.49, 4.00] |
| 20:3n6 | 3.40 | [0.61, 9.00] | 4.41 | [0.60, 18.0] |
| 20:4n6 | 23.1 | [8.00, 51.0] | 28.2 | [5.00, 167] |
| 20:5n3 | 1.41 | [0.13, 4.00] | 1.56 | [0.04, 13.0] |
| 22:4n6 | 0.42 | [0.03, 2.00] | 0.74 | [0.05, 3.00] |
| 22:5n6 | 1.32 | [0.50, 3.00] | 1.01 | [0.11, 4.00] |
| 22:5n3 | 1.15 | [0.12, 3.00] | 2.17 | [0.28, 9.00] |
| 22:6n3 | 10.1 | [3.00, 24.0] | 13.4 | [3.00, 47.0] |
| ***trans-FA*** |  |  |  |  |
| 16:1n7t | 0.76 | [0.18, 2.00] | 2.04 | [0.53, 9.00] |
| 18:2n6tt | 0.28 | [0.03, 1.00] | 0.87 | [0.05, 4.00] |

*†* – Values are reported as geometric means [ranges].
