# Supplementary material for: Type 2 Diabetes Associated Changes in the Plasma Non-Esterified Fatty Acids, Oxylipins and Endocannabinoids
Source: PLoS One. 2012 Nov 8;7(11):e48852. doi: 10.1371/journal.pone.0048852 (PMC3493609; doi:10.1371/journal.pone.0048852)
Supplement: Table S6 — Plasma eighteen carbon oxylipins (nM) in obese African-American women. Geometric mean and ranges are listed for all measured metabolites in this class for experimental groups with and without Type 2 diabetes. (DOC) [file pone.0048852.s006.doc]

**Table S6: Plasma eighteen carbon oxylipins (nM) in obese African-American women *^†^***

| **Compound** | **Parent FA** | **non-diabetic**  **(n=12)** | | **T2D**  **(n=43)** | |
| --- | --- | --- | --- | --- | --- |
| ***Alcohols*** | | | | | |
| 9-HODE | LA | 5.59 | [3.00, 38.0] | 4.94 | [0.01, 38.0] |
| 13-HODE | LA | 16.9 | [4.00, 121] | 21.1 | [3.00, 134] |
| 9-HOTE | ALA | 0.56 | [0.22, 6.00] | 0.49 | [0.06, 3.00] |
| 13-HOTE | ALA | 0.57 | [0.24, 4.00] | 0.47 | [ND, 2.00] |
| ***Ketone*** | | | | | |
| 13-KODE | LA | 5.63 | [3.00, 25.0] | 10.5 | [0.77, 67.0] |
| ***Epoxides*** | | | | | |
| 9(10)-EpOME | LA | 1.18 | [0.34, 11.0] | 2.68 | [0.70, 20.0] |
| 12(13)-EpOME | LA | 1.43 | [0.54, 12.0] | 2.76 | [0.71, 17.0] |
| 12(13)-Ep-9-KODE | LA | 1.69 | [0.80, 18.0] | 2.43 | [0.19, 14.0] |
| 9(10)-EpODE | ALA | 0.32 | [0.08, 4.00] | 0.66 | [0.08, 6.00] |
| 12(13)-EpODE | ALA | 0.07 | [ND, 1.00] | 0.10 | [0.01, 0.92] |
| 15(16)-EpODE | ALA | 0.48 | [0.06, 5.00] | 0.87 | [0.04, 9.00] |
| ***Diols*** | | | | | |
| 9,10-DiHOME | AA | 15.2 | [5.00, 50.0] | 14.3 | [1.00, 51.0] |
| 12,13-DiHOME | AA | 2.08 | [0.59, 6.00] | 1.81 | [0.08, 8.00] |
| 15,16-DiHODE | ALA | 1.35 | [0.45, 4.00] | 1.53 | [0.12, 7.00] |
| ***Triols*** | | | | | |
| 9,10-13-TriHOME | LA | 0.90 | [0.26, 5.00] | 0.74 | [0.06, 3.00] |
| 9,12,13-TriHOME | LA | 2.70 | [0.75, 13.0] | 2.30 | [0.40, 8.00] |

*†* – Values are reported as geometric means [ranges].
