# Supplementary material for: Type 2 Diabetes Associated Changes in the Plasma Non-Esterified Fatty Acids, Oxylipins and Endocannabinoids
Source: PLoS One. 2012 Nov 8;7(11):e48852. doi: 10.1371/journal.pone.0048852 (PMC3493609; doi:10.1371/journal.pone.0048852)
Supplement: Table S7 — Plasma twenty and twenty-two carbon oxylipins (nM) in obese African-American women. Geometric mean and ranges are listed for all measured metabolites in this class for experimental groups with and without Type 2 diabetes. (DOC) [file pone.0048852.s007.doc]

**Table S7: Plasma twenty and twenty-two carbon oxylipins (nM) in obese African-American women *^†^***

| **Compound** | **Parent FA** | **non-diabetic**  **(n=12)** | | **T2D**  **(n=43)** | |
| --- | --- | --- | --- | --- | --- |
| ***Alcohols*** | | | | | |
| 15-HETrE | 20:3n6 | 0.43 | [0.26, 1.00] | 0.44 | [0.05, 1.00] |
| 5-HETE | 20:4n6 | 4.58 | [2.00, 13.0] | 4.01 | [0.61, 21.0] |
| 8-HETE | 20:4n6 | 0.94 | [0.44, 2.00] | 0.85 | [0.01, 4.00] |
| 9-HETE | 20:4n6 | 0.68 | [0.28, 2.00] | 0.66 | [0.07, 3.00] |
| 11-HETE | 20:4n6 | 0.67 | [0.28, 2.00] | 0.54 | [ND, 3.00] |
| 12-HETE | 20:4n6 | 2.56 | [0.94, 4.00] | 3.57 | [0.10, 69.0] |
| 15-HETE | 20:4n6 | 2.15 | [1.00, 9.00] | 1.79 | [0.02, 7.00] |
| 5-HEPE | 20:5n3 | 0.7 | [0.19, 2.00] | 0.63 | [0.05, 3.00] |
| 15-HEPE | 20:5n3 | 0.34 | [0.18, 1.00] | 0.38 | [0.07, 1.00] |
| ***Ketone*** | | | | | |
| 5-KETE | 20:4n6 | 0.32 | [0.14, 2.00] | 0.29 | [0.02, 1.00] |
| 12-KETE | 20:4n6 | 1.3 | [0.12, 10.0] | 1.73 | [0.01, 7.00] |
| 15-KETE | 20:4n6 | 0.69 | [0.34, 3.00] | 0.73 | [0.03, 3.00] |
| ***Epoxides*** | | | | | |
| 8(9)-EpETrE | 20:4n6 | 0.44 | [0.19, 2.00] | 0.56 | [0.11, 3.00] |
| 11(12)-EpETrE | 20:4n6 | 0.46 | [0.23, 2.00] | 0.7 | [0.11, 4.00] |
| 14(15)-EpETrE | 20:4n6 | 0.24 | [0.14, 1.00] | 0.35 | [0.08, 2.00] |
| ***Diols*** | | | | | |
| 5,6-DiHETrE | 20:4n6 | 0.5 | [0.21, 1.00] | 0.45 | [0.06, 2.00] |
| 8,9-DiHETrE | 20:4n6 | 0.35 | [0.11, 0.63] | 0.34 | [0.03, 0.81] |
| 11,12-DiHETrE | 20:4n6 | 0.44 | [0.32, 0.70] | 0.51 | [0.03, 1.00] |
| 14,15-DiHETrE | 20:4n6 | 0.52 | [0.38, 0.72] | 0.49 | [ND, 1.00] |
| 14,15-DiHETE | 20:5n3 | 0.22 | [0.10, 0.45] | 0.23 | [0.01, 0.65] |
| 17,18-DiHETE | 20:5n3 | 1.88 | [1.00, 3.00] | 1.7 | [0.03, 8.40] |
| 19,20-DiHDPA | 22:6n3 | 0.92 | [0.58, 2.00] | 0.97 | [0.03, 7.80] |
| ***Triols*** | | | | | |
| ResolvinE1 | 20:5n3 | 0.59 | [0.09, 2.00] | 0.97 | [0.03, 6.00] |
| ***Prostaglandins*** | | | | | |
| PGF2α | 20:4n6 | 0.64 | [0.24, 1.00] | 0.51 | [0.07, 3.00] |

*†* – Values are reported as geometric means [ranges].
