# Supplementary material for: Type 2 Diabetes Associated Changes in the Plasma Non-Esterified Fatty Acids, Oxylipins and Endocannabinoids
Source: PLoS One. 2012 Nov 8;7(11):e48852. doi: 10.1371/journal.pone.0048852 (PMC3493609; doi:10.1371/journal.pone.0048852)
Supplement: Table S8 — Plasma N-acylethanolamides (nM), lipoamino acids (nM) and monoacylglycerols (µM) in obese African-American women. Geometric mean and ranges are listed for all measured metabolites in this class for experimental groups with and without Type 2 diabetes. (DOC) [file pone.0048852.s008.doc]

**Table S8: Plasma N-acylethanolamides (nM), lipoamino acids (nM) and monoacylglycerols (μM) in obese African-American women *^†^***

| **Compounds** | **Parent FA** | **non-diabetic**  **(n=12)** | | **T2D**  **(n=43)** | |
| --- | --- | --- | --- | --- | --- |
| ***N-Acylethanolamides*** | | | | | |
| P-EA | 16:0 | 9.59 | [5.00, 40.0] | 18.9 | [6.00, 162] |
| S-EA | 18:0 | 16.3 | [2.00, 77.0] | 23.6 | [2.00, 150] |
| O-EA | 18:1n9 | 20.3 | [8.00, 33.0] | 36.2 | [9.00, 175] |
| L-EA | 18:2n6 | 7.91 | [4.00, 27.0] | 11.4 | [5.00, 44.0] |
| DGL-EA | 20:3n6 | 0.50 | [0.30, 0.87] | 0.93 | [0.28, 4.00] |
| A-EA | 20:4n6 | 2.09 | [1.00, 3.00] | 3.57 | [1.00, 8.00] |
| DoP-EA | 22:5n3 | 1.29 | [0.20, 3.00] | 1.96 | [0.68, 5.00] |
| DoHex-EA | 22:6n3 | 0.55 | [0.30, 0.88] | 1.02 | [0.41, 2.00] |
| ***Lipoaminoacids*** | | | | | |
| NO-Gly | 18:1n9 | 8.15 | [2.00, 34.0] | 20.2 | [4.00, 86.0] |
| NA-Gly | 20:4n6 | 0.52 | [0.22, 2.00] | 0.78 | [0.14, 3.00] |
| ***Monoacylglycerols*** | | | | | |
| 1-OG | 18:1n9 | 2.63 | [1.00, 16.0] | 2.73 | [0.58, 22.0] |
| 2-OG | 18:1n9 | 0.44 | [0.11, 4.00] | 0.59 | [0.09, 7.00] |
| 1-LG | 18:2n6 | 0.42 | [0.14, 2.00] | 0.48 | [0.07, 5.00] |
| 2-LG | 18:2n6 | 0.42 | [0.1, 3.00] | 0.53 | [0.07, 8.00] |
| 1-AG | 20:4n6 | 0.12 | [0.04, 0.53] | 0.16 | [0.04, 0.83] |
| 2-AG | 20:4n6 | 0.03 | [0.01, 0.35] | 0.05 | [0.01, 0.40] |

*†* – Values are reported as geometric means [ranges].
